# Community-based surveillance advances the Global Health Security Agenda in Ghana

**DOI:** 10.1371/journal.pone.0237320

**Published:** 2020-08-11

**Authors:** Sharifa Merali, Franklin Asiedu-Bekoe, Alexey Clara, Michael Adjabeng, Isaac Baffoenyarko, Joseph Asamoah Frimpong, Patrick Mawupemor Avevor, Chastity Walker, S. Arunmozhi Balajee

**Affiliations:** 1 Division of Viral Diseases, National Center for Immunization and Respiratory Diseases, Centers for Disease Control and Prevention, Atlanta, Georgia, United States of America; 2 Disease Surveillance Department, Ghana Health Service, Accra, Ghana; 3 Eagle Medical Services LLC, Contracting Agency to the Division of Viral Diseases, National Center for Immunization and Respiratory Diseases, Centers for Disease Control and Prevention, Atlanta, Georgia, United States of America; 4 Division of Global Health Protection/Ghana, Center for Global Health, Centers for Disease Control and Prevention, Atlanta, Georgia, United States of America; 5 World Health Organization, Ghana Country Office, Accra, Ghana; The University of Hong Kong, CHINA

## Abstract

Ghana Health Service (GHS) has strengthened community-based surveillance (CBS) to facilitate early detection and rapid reporting of health events of all origins. Since June 2017, GHS has employed an event-based surveillance approach at the community level in a phased manner. CBS coverage has broadened from 2 to 30 districts across Ghana. Through this effort, capacity was built across all administrative levels in these districts to detect, report, triage, and verify signals, and to perform risk assessment and investigate events. Data were collected and analyzed during an evaluation of initial 2-district implementation in March 2018 and during expanded 30-district implementation in March 2019. Between September 2018 and March 2019, 317 health events were detected through CBS. These events included vaccine-preventable disease cases, acute hemorrhagic conjunctivitis outbreaks, clusters of unexpected animal deaths, and foodborne illness clusters. Eighty-nine percent of the 317 events were reported to district-level public health staff within 24 hours of detection at the community level, and 87% of all detected events were responded to within 48 hours of detection. CBS detected 26% of all suspected vaccine-preventable disease cases that were reported from implementing districts through routine disease surveillance. GHS strengthened CBS in Ghana to function as an early warning system for health events of all origins, advancing the Global Health Security Agenda.

## Introduction

The Global Health Security Agenda (GHSA) was launched in 2014 [1]. GHSA aims to strengthen countries’ capacity to prevent, detect, and respond to emerging public health threats in a manner that aligns with International Health Regulations requirements [1, 2]. Ghana officially committed to the GHSA in 2015, and began to strengthen a number of areas, including surveillance [3]. Ghana’s most recent Joint External Evaluation (JEE) in February 2017 revealed gaps in its real-time surveillance capacity [2]. The community-based surveillance (CBS) system was functioning sub-optimally [2]. One Health (i.e., human-animal-environment health interactions) principles were poorly integrated into community-level surveillance; CBS Volunteers (CBSVs) had limited knowledge of and support for animal health surveillance, and there were few reporting linkages between human and animal health sectors [2]. Capacity for event-based surveillance (EBS) was limited at all administrative levels [2].

In Ghana, surveillance information is collected and reported from health facilities and hospitals through case-based or syndromic data collection, following the Integrated Disease Surveillance and Response (IDSR) framework adapted from the World Health Organization Regional Office for Africa [4]. CBS was established as a component of IDSR in 1998, following the successes of the Guinea Worm Eradication Program [5, 6]. Community engagement and active surveillance by CBSVs were essential for Guinea worm eradication, and more broadly, for surveillance in the country [5, 6].

EBS is the “organized collection, monitoring, assessment, and interpretation of mainly unstructured ad-hoc information regarding health events or risks, which may represent an acute risk to human health” [7, 8]. Information is collected as signals, defined as “data and/or information representing a potential acute risk to human health, and may consist of reports of cases or deaths (individual or aggregated), potential exposure of human beings to biological, chemical, or radiological and nuclear hazards, or occurrence of natural or man-made disasters” [7, 8]. Signals can be detected through any potential source, including community-level sources like CBS. Because of the high sensitivity of EBS, once reported, signals must be triaged and verified to filter out false reports, confirm authenticity of the possible event, and characterize its nature [7]. Once verified, a signal becomes an event [7, 8]. Signal triage and verification ensure the public health system is not unduly burdened [7].

The One Health approach can address “urgent, ongoing, or potential health threats at the human-animal-environment interface at sub-national, national, global, and regional levels”, and requires balance and collaboration among multiple relevant disciplines and sectors [9]. EBS and One Health are complementary: EBS can enhance One Health through rapid detection of a wide range of events occurring among and between humans, animals, and the environment, including zoonotic diseases; One Health can enhance EBS by providing a platform for coordinating surveillance and information sharing across all relevant sectors so that health events can be addressed in a harmonized manner [9].

Ghana Health Service (GHS) began a project in June 2017 to address gaps revealed by the JEE. This resulted in implementation of modified CBS in several districts over a period of two years. Here we describe the modified CBS system in Ghana, including the design and implementation processes, as well as lessons learned and gaps revealed during implementation.

## Materials and methods

In Ghana, IDSR data collection and reporting occur within existing health administrative structures [4]. At the lowest administrative level, data are collected by public health staff in Community-based Health Planning and Services (CHPS) Zones; aggregated data are reported up to sub-districts, and then to districts. District-level public health authorities in turn analyze data from their catchment areas, enter the data into an electronic database, and initiate response actions as required [4]. GHS public health authorities at regional and national levels have access to these surveillance data, and are sent reports on a regular basis [4]. CBS utilizes CBSVs to detect and report selected priority diseases and unusual health events to the CHPS Zones, who then follow established mechanisms for investigation, recording, reporting, and response through IDSR [4, 10].

**Fig 1:**
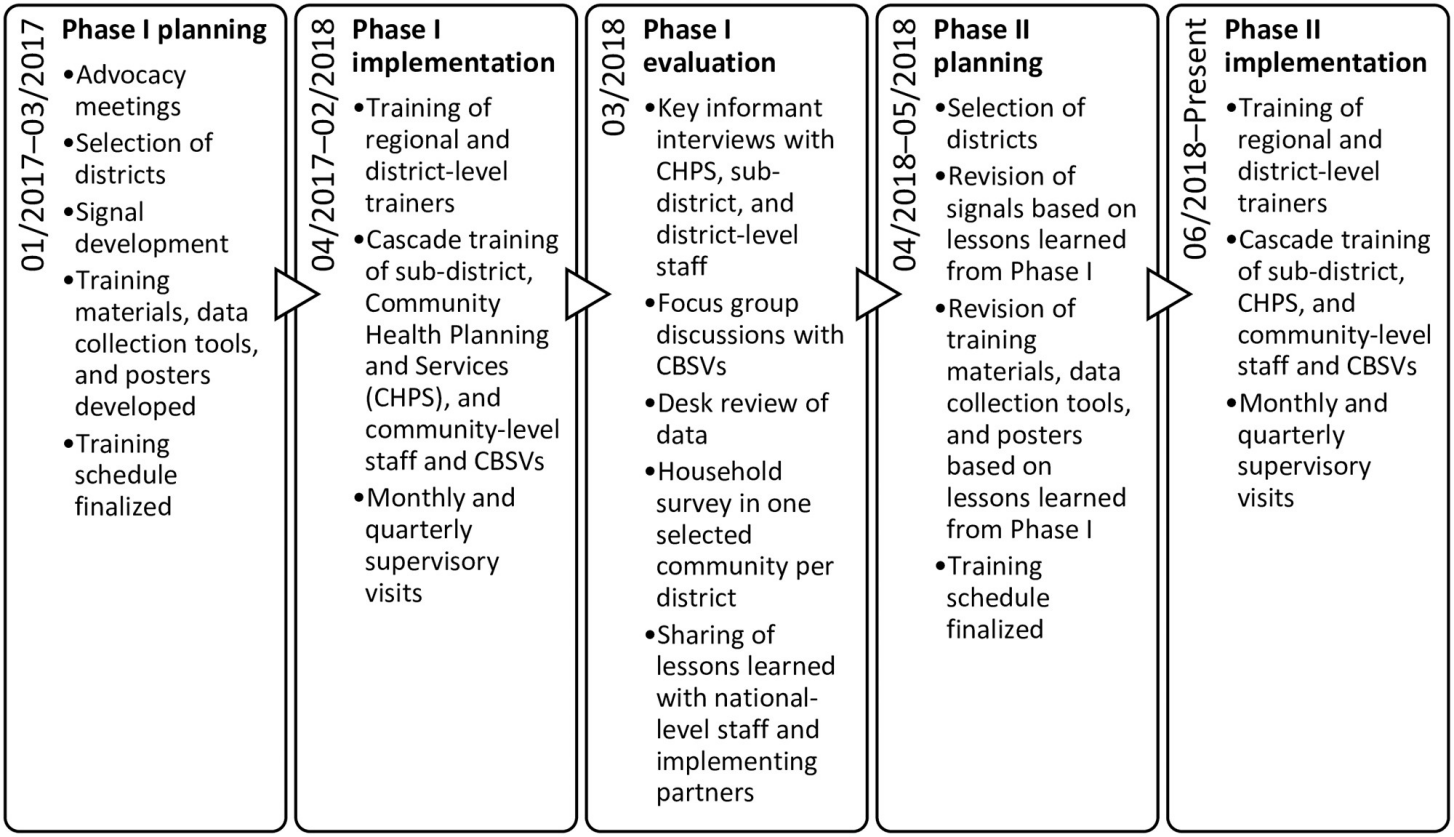
Timeline of Phase I and II modified CBS activities in Ghana.

Implementation of modified CBS in Ghana occurred in two phases. Phase I was piloted in two districts in June 2017 and evaluated in March 2018. Lessons learned from Phase I informed Phase II of modified CBS. Phase II was implemented in 30 districts across Ghana, including the two districts from Phase I, in June 2018. These activities were led by GHS in collaboration with the United States Centers for Disease Control and Prevention (CDC), the International Organization for Migration (IOM), and the WHO Ghana Country Office. A timeline of all Phase I and II modified CBS activities in Ghana is illustrated in Fig 1.

### Phase I implementation of modified CBS

**Fig 2:**
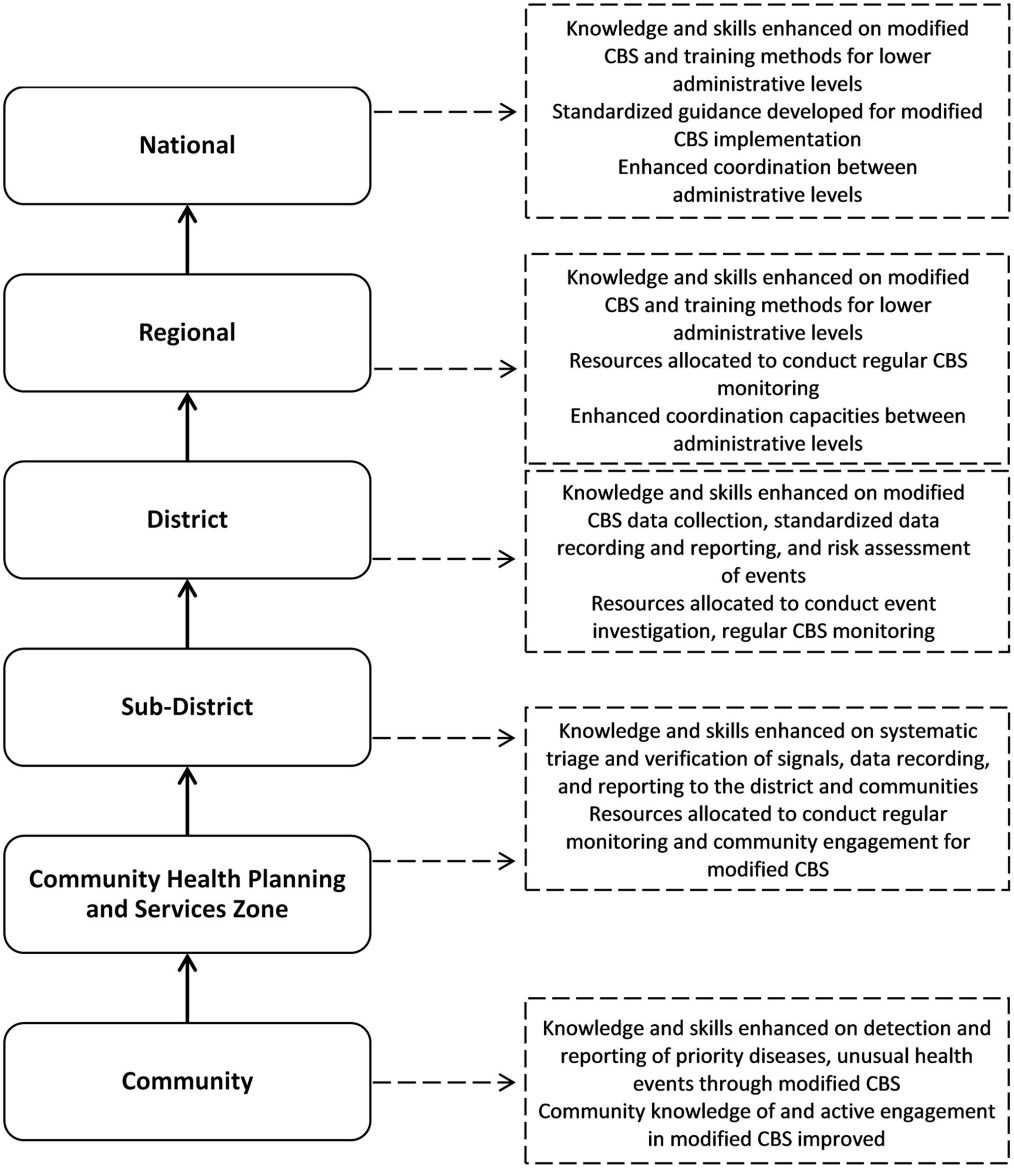
Surveillance and reporting structure in Ghana (left); capacities improved through modified CBS at each administrative level shown in dashed boxes (right).

Planning for Phase I implementation of modified CBS in Ghana began with meetings in January 2017 between GHS, the Veterinary Services Directorate (VSD) within Ghana’s Ministry of Food and Agriculture, CDC, IOM, and WHO. During these meetings, stakeholders discussed how the existing CBS program would be modified and integrated into the existing reporting and surveillance structure (Fig 2). As a result of these discussions, five major implementation elements were identified and agreed upon. First, two districts were selected for Phase I implementation: Ketu South, a peri-urban district bordering Togo, and Kassena Nankana West, a rural district bordering Burkina Faso. Second, a list of signals for detection of unusual health events, including events related to animal health, was drafted based on public health risks identified by GHS and VSD (S1 Table). Third, an incentive package that all CBSVs would receive as part of the implementation was defined. Fourth, a timeline for training and implementation was agreed upon. Finally, tools and materials were developed for Phase I implementation, complementing existing materials used for CBS in Ghana (S2 Table).

In March 2017, GHS district-level surveillance officers from the two districts and their regional counterparts were trained on all aspects of modified CBS: signal detection, reporting, use of data collection tools, roles and responsibilities, and supervision. Additionally, training was provided on the coordination necessary between GHS and VSD at the CHPS Zone and community levels to facilitate rapid reporting and investigation of animal health events. Regional and district-level officers were also taught how to conduct training for staff at the sub-district and CHPS Zone levels, as well as for CBSVs. In May 2017, district and regional level trainers trained sub-district staff, CHPS Zone staff, and CBSVs in their respective districts. In June 2017, the two selected districts officially launched modified CBS.

During Phase I implementation, districts submitted modified CBS data on a monthly basis to their regional and national counterparts via email, using Microsoft Excel spreadsheets. Regional and district-level surveillance officers conducted supervisory visits on a regular basis in accordance with Ghana’s IDSR Guidelines [4].

### Phase I evaluation

In March 2018, after 10 months of implementation, GHS evaluated modified CBS in both districts, with technical assistance from CDC, IOM, and WHO. One quantitative and three qualitative data collection tools were utilized in this Phase I evaluation. A data extraction tool was used to quantitatively extract signal and event data recorded at the district level from the beginning of Phase I implementation in June 2017. This data was analyzed in Microsoft Excel. Three qualitative data collection tools—acceptability surveys, key informant interviews, and focus group discussions—were employed at all implementing administrative levels to identify key themes, including acceptability of modified CBS among staff, usefulness, barriers to implementation, and direct and indirect perceived impacts of implementation. In addition to these tools, a household survey was employed at the community level in an attempt to assess CBSV effectiveness to detect signals emerging in their communities.

### Phase II implementation of modified CBS

Challenges and subsequent solutions identified through Phase I evaluation informed Phase II planning and implementation. Planning for Phase II of modified CBS took place from April to May 2018. Signals were revised to increase specificity of detection at the community level, and a signal was added to capture occurrence of suspected yellow fever in communities (S1 Table). Data collection tools and posters were revised to reflect the revised signals (S2 Table). The incentive package for CBSVs was also revised based on lessons learned from the Phase I implementation and to plan for future sustainability. Ghana Health Service selected 28 more districts where modified CBS would be implemented, prioritizing rural districts with hard-to-reach communities whose residents might face difficulty in accessing healthcare.

The methods for training, data collection, and supervision of Phase II of modified CBS were similar to that of Phase I. Two training workshops were held for regional and district-level GHS staff: the first took place in May 2018 for selected districts in northern Ghana, while the second took place in June 2018 for selected districts in southern Ghana. Between July and September 2018, training took place in each implementing district using the same approach as in Phase I. To facilitate rapid data collection and analysis of summarized CBS data, GHS designed a Google Sheet© that all implementing districts are required to complete on a monthly basis.

In April 2019, after 7 months of Phase II modified CBS implementation, data were collectively analyzed across all 30 districts to understand their progress and address any challenges that arose.

### Analysis of Phase II modified CBS data

For this study, each reported incident captured at the community level—whether existing lay case definitions in IDSR for priority diseases, or signals denoting unusual health events developed as part of this initiative—was labelled a “signal.” All signals were triaged as relevant for early warning purposes; those that were verified as having truly occurred (i.e., not a false alarm) were termed “events.”

Data on all signals and events captured and analyzed through Phase II modified CBS were gathered from September 2018 to March 2019. The event-to-signal ratio, a metric of specificity, was calculated by dividing the number of events reported by the number of signals detected by month. Based on the observed trend of signal detection over time, a Chi-square test was carried out, assuming that the signals detected were Poisson variates. The total number of signals detected in the October–December 2018 three-month time period was compared to the total number of signals detected in the January–March 2019 three-month time period to evaluate the difference in signal detection between both time frames. Similarly, the mean event-to-signal ratio in the October–December 2018 three-month time period was compared to the mean event-to-signal ratio in the January–March 2019 three-month time period. A *p*-value was established for each comparison, with *p*<0.05 as the threshold for statistical significance. Events were assigned to one of five categories based on their characteristics—animal-related events, vaccine-preventable diseases (VPDs), foodborne illnesses, other infectious diseases, and adverse event(s) following immunization—as well as the type of signal used for detection. Timeliness of the system was assessed using two timeframes for events detected: (1) time from detection at the community level to notification at the district level and (2) time from detection at the community level to response initiation, as recorded at the district level. Timeliness was calculated in days, and the following calculations were done for each respective timeframe: percentage of events reported to the district level within 24 hours and percentage of events responded to within 48 hours of detection. Finally, the percentage of cases reported to IDSR that were detected by CBS was calculated for each region with implementing districts. Cases included four selected VPDs: acute flaccid paralysis (AFP), measles, yellow fever, and meningitis. These priority diseases were selected because suspected case reports were collected through both IDSR and modified CBS. All analyses were conducted in Microsoft Excel 10.0.

## Results

### Phase I implementation of modified CBS

Phase I of modified CBS covered 2 districts, 166 communities, and 1% of the total population of Ghana. A total of 96 personnel and 404 CBSVs were trained on the functions of CBS, reaching 1.4 CBSVs per 1,000 population. An incentive package was also provided to each CBSV (Table 1). Major challenges from the Phase I evaluation and corresponding solutions for Phase II implementation were identified and employed, respectively (S3 Table).

**Table 1:** Implementation elements of modified CBS in Phase I and Phase II.

Implementation Element	Phase I	Phase II
# of districts	2	30
# of subdistricts	15	162
# of CHPS Zones	52	577
# communities	166	3,503
Population covered by modified CBS	279,418	2,541,713
# personnel trained	96	706
# CBSVs trained	404	3,930
# CBSVs trained per 1,000 population	1.4	1.5
# minimum incentive packages distributed	404	3,930

### Phase II implementation of modified CBS

In Phase II, 28 more districts—encompassing 3,337 communities—were added, increasing modified CBS coverage to 9% of the total population of Ghana. A total of 706 staff and 3,930 CBSVs were trained. A revised incentive package was distributed to CBSVs in the 28 added districts (Table 1).

**Fig 3:**
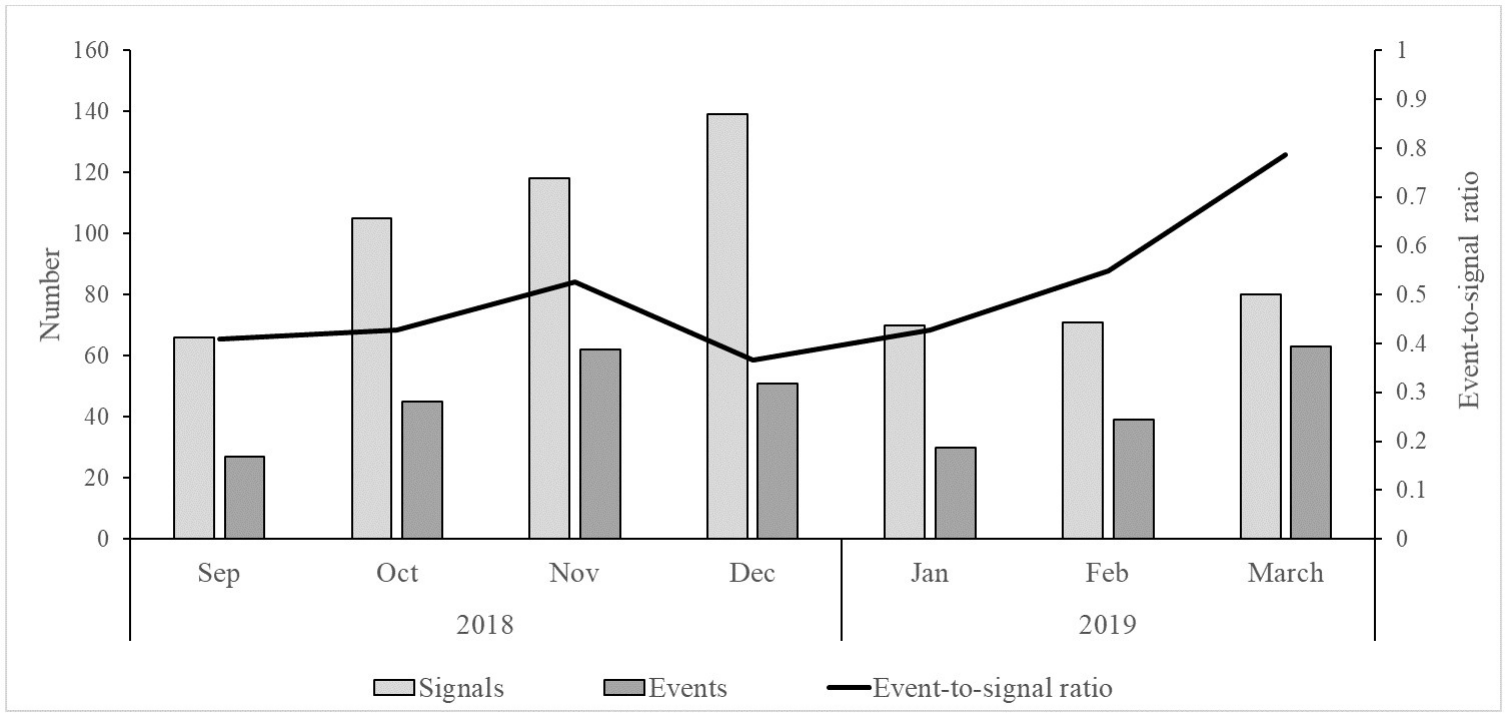
Number of signals and events reported, by month, from Phase II modified CBS districts, September 2018–March 2019.

From September 2018 to March 2019, 649 signals were detected; 317 (49%) were verified as health events. The number of signals detected showed an increasing trend from September to December 2018, after which it decreased, remaining stable through March 2019. The number of signals detected in the three-month period from January to March 2019 was found to be significantly lower (*p*<0.001) than the number of signals detected in the three-month period from October to December 2018 (S4 Table). The event-to-signal ratio by month ranged from 0.43 to 0.37 from October to December 2018, and increased from January 2019 onwards, reaching 0.79 in March 2019 (Fig 3). However, there was no significant difference (*p* = 0.26) between the mean event-to-signal ratio in the three-month time period from October to December 2018 (0.44) as compared to the three-month time period from January to March 2019 (0.59).

Of the 317 events reported, 156 (49%) were animal-related events, including suspected rabies and other animal bites and unexpected animal deaths; 134 (42%) were VPD cases including AFP, chickenpox, and suspected cases of measles, yellow fever, and meningitis. Thirteen foodborne illness events, four acute hemorrhagic conjunctivitis outbreaks, one cluster of infectious arthritis cases, and one cluster of skin disease cases were detected through modified CBS (Table 2).

**Table 2:** Type of events detected during Phase II modified CBS implementation, September 2018–March 2019.

Events detected	Number	%
***Animal-related events***	**156**	**49**
Suspected rabies and other animal bites	150	47
Unexpected animal deaths	6	2
***Vaccine-preventable diseases***	**134**	**42**
Suspected measles	77	24
Suspected yellow fever	25	8
Acute flaccid paralysis	20	6
Suspected meningitis	10	3
Chickenpox	2	<1
***Foodborne illnesses***	**13**	**4**
***Other infectious diseases***	**13**	**4**
Acute hemorrhagic conjunctivitis	4	1
Malaria	4	1
Skin diseases	3	<1
Suspected cholera	1	<1
Infectious arthritis	1	<1
***Adverse event following immunization***	**1**	**<1**
**Total**	**317**	**100**

Events detected between September 2018 and March 2019 by each signal used during Phase II modified CBS implementation were described (S5 Table). The signals “any person with fever and rash” and “two or more persons with similar severe illnesses in the same setting within 1 week” facilitated the reporting of the most diverse events. All six unexpected animal death events were detected through the signal “any event in the community that causes public anxiety.” Sixty-four percent (202/317) of all events were detected using the signals developed as part of this modified CBS implementation, including all reported animal-related events (156), acute hemorrhagic conjunctivitis outbreaks (4), and single suspected yellow fever cases.

Timeliness data were available for 176 (56%) of the 317 events. The time from community-level detection to district-level notification and time from community-level detection to response initiation were <24 hours and <48 hours, respectively, for 86% of animal-related events. Ninety-one percent of all suspected measles cases were reported to the district level within 24 hours of detection at the community level, while 90% were responded to within 48 hours of detection. All AFP cases were reported to the district level and responded to within 24 and 48 hours, respectively (Table 3).

**Table 3:** Time from community-level detection to district-level notification and response initiation of events reported from September 2018–March 2019 (N = 176).

Type of event	No. of events	No. (%) of events that were notified within 24 hours after detection	No. (%) of events that were responded to within 48 hours after detection
Animal-related events	88	76 (86)	76 (86)
Suspected measles	58	53 (91)	52 (90)
AFP	11	11 (100)	11 (100)
Others*	19	17 (89)	14 (74)
**Total**	**176**	**157 (89)**	**153 (87)**

*Other events include eight suspected meningitis cases, six suspected yellow fever cases, two foodborne illness events, two cases of malaria, and one outbreak of acute hemorrhagic conjunctivitis.

Response data were available for 235 (74%) of the 317 events detected during Phase II of modified CBS implementation (S1 Fig). Several activities conducted as part of response were registered for each event. Clinical care was provided in 82 (91%) of animal-related events and in 49 (37%) of VPD events. Appropriate specimens for laboratory testing were collected for 126 (94%) of all VPD cases. Laboratory result data was not recorded for 98% (123) of these 126 VPD cases. General community sensitization and health education for affected persons were carried out in 50 (55%) and 33 (37%) of animal-related events, respectively. These same activities were conducted in 14 (10%) and 20 (15%) of VPD events, respectively. Coordination with the animal sector was registered as a response activity for two animal-related events.

The percentage of VPD cases reported to IDSR that were detected through modified CBS varied by region, ranging from 0% to 61%, with an overall percentage of 26%. Districts implementing modified CBS in the Ashanti region had the highest percentage (61%). Implementing districts in the Greater Accra, Western, and Upper East regions did not report any VPD cases through modified CBS during Phase II implementation (Table 4).

**Table 4:** Number and percentage of cases of VPDs captured in IDSR that were detected through modified CBS, September 2018–March 2019.

Region	Cases of VPDs* captured in IDSR, No.	Cases of VPDs reported in IDSR that were detected through CBS, No. (%)
Ashanti	18	11 (61)
Volta	41	16 (39)
Upper West	105	35 (33)
Eastern	35	11 (31)
Central	17	5 (29)
Brong Ahafo	42	7 (17)
Northern	77	2 (3)
Greater Accra, Western, Upper East	92	**0 (0)**
**Total**	**335**	**87 (26)**

*VPDs: acute flaccid paralysis, suspected measles, suspected meningitis, and suspected yellow fever.

## Discussion

This study demonstrates that modified CBS allows for early detection and reporting of diverse public health events in Ghana. Data from both phases of implementation show that modified CBS detected more than 300 events that might otherwise have been missed by the routine surveillance system. Over half of all events detected were either animal health events or foodborne illnesses, neither of which are captured by IDSR. Approximately one-quarter of all VPD cases reported were detected through modified CBS, and in some districts the contribution of modified CBS reached up to two-thirds of total reports.

The modified CBS system attempted to incorporate a One Health approach at the community level. CBSVs were trained to detect and report both human and animal health events, as well as to communicate and coordinate with their VSD counterparts to follow up on animal health events detected in their communities. The VSD was directly involved in all aspects of modified CBS, including design and revision of signals and the sensitization and involvement of veterinary officers in the system. This collaboration between GHS and VSD resulted in the detection of and response to >150 animal-related events and unexpected animal deaths over the course of Phase II implementation. The modified CBS system also enabled timely (<24 hours) notification of several local health events and outbreaks that resulted in prompt response by public health authorities.

Experience implementing modified CBS in Ghana resulted in several lessons learned. First, the design of signals to strike a balance between sensitivity and specificity is paramount for an effective CBS system. Signals designed to be very sensitive may yield information that is not relevant for public health; for instance, in Phase I, the signal, “any person who has been bitten, scratched, or whose wound has been licked by a dog, cat, or other animal” resulted in reporting of a large number of signals, of which only half qualified as events. When this signal was re-designed to be narrower in scope, the event-to-signal ratio improved. Similarly, adding a timeframe (i.e., 1 week) for occurrences of school absenteeism made this signal more specific. At the same time, signals designed to recognize patterns, such as clusters, might have to be broad to detect diverse type of events. For example, the signal “two or more persons with similar severe illnesses in the same setting within 1 week” enabled the detection of the most diverse events of all signals. In this study, some signals did not result in reporting of any events. It is not clear whether these occurrences are rare and/or not easily captured by community health workers. Regardless, the list of signals should be considered dynamic—a list that is periodically reviewed and adjusted after systematic program evaluation and/or to fit a surveillance need. Second, the inclusion of a risk assessment training for the district-level public health staff allowed for correct triaging and verification of reported signals, reducing background noise from a highly sensitive surveillance system. Lastly, when CBS programs are planned, careful consideration should be given to the design of data collection and reporting tools. These tools should aim to collect a minimum set of data that can provide usable information, should be clear and simple, and minimize burden to implementers. These tools should also be designed to provide data that support monitoring and evaluation of these programs.

There were several limitations in this study. The study found that the event-to-signal ratio increased from December 2018 to March 2019; however, it was not possible to determine the reason for this increase during this study period. A similar effect was seen in CBS implementation in Vietnam [11] but needs to be further understood. This study was not designed to collect data on the effect of seasonality of certain diseases, vaccine campaigns, or other trends in incidence and reporting of health events. Although the data collected over the course of Phase II showed no spikes or aberrations indicating such an impact for any particular event, this should be further explored in future studies. Another limitation of this study was the difficulty in measuring the impact of modified CBS. The electronic database used for the recording of surveillance data through IDSR does not allow for the recording of clusters of illnesses, clusters of deaths, nor does it link to animal or environmental health surveillance data. Generally, unusual health events detected through CBS are recorded on paper at the district level and are not integrated into existing routine surveillance databases. Moreover, CBS data recorded in IDSR databases are indistinguishable from data from health facilities, limiting the ability to gauge the contributions of modified CBS from this data source. Data recorded in paper-based logbooks used at the district level are not consolidated into any central database. With continued inconsistencies in data recording, these logbooks are a weak gold standard of comparison, as all previously occurring outbreaks detected at the community level may not have been recorded in a standardized manner. GHS is currently reviewing how the IDSR database can be made more compatible for recording event data, particularly clusters of cases, as well as to better record events and priority diseases captured through CBS. Standardized and consistent recording of event data, particularly related to timeliness and response activities, continued to be a challenge in both phases of the study. Though the data quality from Phase II implementation of modified CBS was markedly higher than that of Phase I, just slightly more than half of all events had time stamps and response activities recorded. As a result, it is important to consider that the timeliness data presented in this paper could be biased, as there was no information available for 141 events detected during the timeframe of analysis. This limitation is currently being addressed by GHS staff by reinforcing the importance of proper record maintenance during supervisory visits at the district level. Response activities that were recorded were not documented systematically, making it challenging to extract information about them. Response activities describing coordination with VSD for animal health events, for example, were very limited, despite the emphasis placed on this during training. While these activities may have been recorded in animal health surveillance databases, the lack of formalized reporting linkages between human and animal health prevents their accessibility. This challenge was previously identified in the findings of Ghana’s JEE in 2017; more needs to be done to build formalized reporting linkages between human and animal health surveillance in Ghana [2]. Finally, regional differences in the contribution of modified CBS to IDSR to detect priority diseases may be due to differences in engagement, training application, and available resources.

Despite the limitations and challenges in implementing CBS, GHS demonstrated that this approach can contribute to the early warning function of IDSR, improve One Health linkages, and help to advance the GHSA overall. GHS must continue to address the challenges identified in this study to improve the modified CBS system.

Ghana’s approach to implementing CBS has informed regional and global guidance. In 2018, the Africa Centres for Disease Control and Prevention published a framework for EBS, consulting with Member States including Ghana, to provide guidance and best practices for countries who wish to establish EBS [12]. In 2019, WHO published a meeting report describing how CBS should be defined, implemented, and strengthened, based on various countries’ experiences, including that of Ghana [13]. This approach has also been implemented, or is currently being implemented, in a number of countries, including Kenya, Cameroon, Côte d’Ivoire, Tanzania, Vietnam, Senegal, and India.

## Supporting information

S1 Table Signals used in Phase I and II modified CBS implementation. (DOCX)

S2 Table Tools utilized at each administrative level for Phase I and Phase II modified CBS implementation in Ghana. (DOCX)

S3 Table Challenges from evaluation of Phase I modified CBS implementation in Ketu South and Kassena Nankana West districts, and solutions implemented for Phase II. (DOCX)

S4 Table Number of signals and events detected, by month, from Phase II modified CBS districts, September 2018 –March 2019. (DOCX)

S5 Table Type of events detected by each signal used during Phase II modified CBS implementation, September 2018–March 2019. (DOCX)

S1 Fig Type of response activities carried out for events detected through Phase II modified CBS implementation from September 2018–March 2019 (N = 239). (DOCX)
