# Supplementary material for: Community-based surveillance advances the Global Health Security Agenda in Ghana
Source: PLoS One. 2020 Aug 11;15(8):e0237320. doi: 10.1371/journal.pone.0237320 (PMC7418973; doi:10.1371/journal.pone.0237320)
Supplement: S1 Table — (DOCX) [file pone.0237320.s001.docx]

**S1 Table. Signals used in Phase I and II modified CBS implementation.**

| **Signals used from the existing CBS** | **Signals added for Phase I implementation** | **Revisions to signals added in Phase I, for Phase II implementation** |
| --- | --- | --- |
| - Any person with fever and neck stiffness - Any person who developed sudden weakness in the limbs - Any person with worms emerging from any part of the body - Any person with fever and rash - Any newborn who is able to suck and cry at birth and then, after 2 days, is unable to suck or feed and becomes stiff - Any person 5 years of age or more with lots of watery diarrhea and sometimes vomiting profusely as well. In the case of an outbreak, anybody who passes watery/loose stool. | - Two or more persons with similar severe illnesses in the same setting within 1 week | - No revision |
|  | - Two or more persons dying in the same community within 1 week | - No revision |
|  | - Any human illness or death after exposure to animals and animal products, including poultry | - No revision |
|  | - Two or more persons that pass watery stools and/or vomiting after eating/drinking at a given setting | - No revision |
|  | - Any event in the community that causes public anxiety | - No revision |
|  | - Any person who has been bitten, scratched, or whose wound has been licked by a dog, cat, or other animal | - Any person who has been bitten by a stray or sick dog, cat, or other animal |
|  | - Unexpected large numbers of children absent from school due to the same illness | - Unexpected large numbers of children absent from school due to the same illness within 1 week |
|  | - Increase in number of animal sicknesses and/or deaths, including poultry, within 1 week | - Signal removed |
|  |  | - Signal added: Any person with fever and yellowish discoloration of the eyes |
