# Supplementary material for: Community-based surveillance advances the Global Health Security Agenda in Ghana
Source: PLoS One. 2020 Aug 11;15(8):e0237320. doi: 10.1371/journal.pone.0237320 (PMC7418973; doi:10.1371/journal.pone.0237320)
Supplement: S2 Table — (DOCX) [file pone.0237320.s002.docx]

**S2 Table. Tools utilized at each administrative level for Phase I and Phase II modified** **CBS implementation in Ghana.**

| **Administrative Level** | **Tools utilized in CBS implementation** |
| --- | --- |
| Regional and national | CBS Guidelines**  CBS Training Manual* |
| District | CBS Guidelines**  CBS Training Manual*  District Logbook of Rumours and Suspected Outbreaks**  District CBS Summary Form*  CBS Monitoring Checklists |
| Sub-district | Sub-District CBS Summary Form*  CBS Monitoring Checklists |
| CHPS Zone | CHPS Community Surveillance Reporting Logbook*  Verification Tool*  CHPS CBS Summary Form*  CBS Poster*  CBS Monitoring Checklists |
| Community | CBS Register**  CBS Poster* |

* Tool revised following Phase I implementation.

** Existing tool from Ghana IDSR, modified for implementation.
