# Supplementary material for: Community-based surveillance advances the Global Health Security Agenda in Ghana
Source: PLoS One. 2020 Aug 11;15(8):e0237320. doi: 10.1371/journal.pone.0237320 (PMC7418973; doi:10.1371/journal.pone.0237320)
Supplement: S3 Table — (DOCX) [file pone.0237320.s003.docx]

**S3 Table. Challenges from evaluation of Phase I modified CBS implementation in Ketu South and Kassena Nankana West districts, and solutions implemented for Phase II.**

|  | **Major challenge from Phase I** | **Solution implemented for Phase II** |
| --- | --- | --- |
| 1 | Sensitivity of signals designed to capture unusual health events was very high, resulting in the reporting of many non-events | Revise signals to increase the specificity of unusual health event detection |
| 2 | Inconsistent and non-standardized recording of event details at the district level, resulting in poor data quality | Revise tools to increase clarity  Emphasize correct usage of data collection tools during training for Phase II implementation |
| 3 | CBSVs provided with incentive package that may not be sustainable | Adjust incentive package to a sustainable level while maintaining productivity and engagement at the community level |
| 4 | High health-seeking behaviors among residents in communities with easy access to healthcare facilities, mostly in peri-urban areas, resulting in weaker engagement with CBSVs | Districts selection for Phase II implementation of modified CBS should focus on areas with low access to healthcare to ensure strong engagement with CBSVs |
