# Supplementary material for: Community-based surveillance advances the Global Health Security Agenda in Ghana
Source: PLoS One. 2020 Aug 11;15(8):e0237320. doi: 10.1371/journal.pone.0237320 (PMC7418973; doi:10.1371/journal.pone.0237320)
Supplement: S4 Table — (DOCX) [file pone.0237320.s004.docx]

**S4 Table. Number of signals and events detected, by month, from Phase II modified** **CBS districts, September 2018 – March 2019.**

| **Month** | **Signals detected (no.)** | **Events detected (no.)** |
| --- | --- | --- |
| September | 66 | 27 |
| October | 105 | 45 |
| November | 118 | 62 |
| December | 139 | 51 |
| January (2019) | 70 | 30 |
| February | 71 | 39 |
| March | 80 | 63 |
