# Supplementary material for: Community-based surveillance advances the Global Health Security Agenda in Ghana
Source: PLoS One. 2020 Aug 11;15(8):e0237320. doi: 10.1371/journal.pone.0237320 (PMC7418973; doi:10.1371/journal.pone.0237320)
Supplement: S5 Table — (DOCX) [file pone.0237320.s005.docx]

**S5 Table. Type of events detected by each signal used during Phase II modified CBS implementation, September 2018–March 2019.**

| **Signal** | **Type(s) of events detected (No.)** |
| --- | --- |
| Any person with fever and neck stiffness | Suspected meningitis (10)  Malaria (2) |
| Any person who developed sudden weakness in the limbs | AFP (20) |
| Any person with worms emerging from any part of the body | - |
| Any person with fever and rash | Suspected measles (77)  Chickenpox (2)  Skin diseases (2)  Malaria (1) |
| Any newborn who is able to suck and cry at birth and then, after 2 days, is unable to suck or feed and becomes stiff | - |
| Any person 5 years of age or more with lots of watery diarrhea and sometimes vomiting profusely as well. In the case of an outbreak, anybody who passes watery/loose stool. | Suspected cholera (1) |
| Two or more persons with similar severe illnesses in the same setting within 1 week | Animal bite (1)  Infectious arthritis (1)  Acute hemorrhagic conjunctivitis (3)  Adverse event following immunization (1) |
| Two or more persons dying in the same community within 1 week | - |
| Any human illness or death after exposure to animals and animal products, including poultry | - |
| Two or more persons that pass watery stools and/or vomiting after eating/drinking at a given setting | Foodborne illnesses (13) |
| Any person who has been bitten by a stray or sick dog, cat, or other animal | Suspected rabies (110)  Other animal bites (39) |
| Unexpected large numbers of children absent from school due to the same illness within 1 week | Acute hemorrhagic conjunctivitis (1) |
| Any event in the community that causes public anxiety | Unexpected animal deaths (6)  Skin disease (1) |
| Any person with fever and yellowish discoloration of the eyes | Suspected yellow fever (25)  Malaria (1) |
